# 

**Published:** 1907-11-09

**Authors:** 


W. W. Bell, L.S.A., has been appointed Clinical Assistant to St. John's Hospital for Diseases of the Skin, Leicester Square, W.C.

### POST-GRADUATE DIARY FOR NEXT WEEK.

The Post-Graduate College, West London Hospital, Hammersmith, W.—Daily at 2, Medical and Surgical Clinics; at 2.30, Operations. Special Departments at 10 (Tuesday, Wednesday, Friday), and daily at 2. Lectures, at 5 p.m. :—November 11, Dr. Saunders, Clinical; November 12, Mr. Pardoe, Treatment of Vesical Calculus; November 13, Dr. Beddard, Practical Medicine; November 14, Mr. Keetley, Injuries and Diseases of the Foot; November 15, Dr. Cole, Pathology of General Paralysis. At 12 noon, November 11, Dr. Low, Pathological Demonstration. Practical Classes and Demonstrations daily.

Medical Graduates' College and Polyclinic, Chenies Street, W.C.—Clinics, Demonstrations and Practical Classes daily, except Saturdays. Lectures at 5.15 p.m. :—November 11, Mr. Gunn, Eyesight in Old Age; November 12, Dr. Raw, Human and Bovine Tuberculosis; November 13, Mr. Armour, Head Injuries; November 14, Mr. Jessett, Carcinoma of the Uterus. The Christmas Session of Practical Classes began on November 4.

London School of Clinical Medicine, Seamen's Hospital, Greenwich.—Medical and Surgical Clinics, Demonstrations, and Special Departments daily. Lectures :—November 12, 3.15 p.m., Mr. Carless; November 14, 2.15 p.m., Dr. Rankin, Infantile Paralysis.

North-East London Post-Graduate College, Prince of Wales' Hospital, Tottenham, N.—Surgical, Medical, and Special Clinics daily, Saturdays excepted. Special Lectures and Demonstrations :—November 13, 4.30 p.m., Mr. Brooks, Eye Cases; November 14, 5 p.m., Dr. Squire, Chest Cases (at Mount Vernon Hospital, Hampstead, N.W.).

The West End Hospital for Diseases of the Nervous System, Welbeck Street, W.—At 5 p.m., November 14, Dr. Dundas Grant, Some Forms of Nerve Deafness.

